# Molybdenum Diselenide and Tungsten Diselenide Interfacing Cobalt-Porphyrin for Electrocatalytic Hydrogen Evolution in Alkaline and Acidic Media

**DOI:** 10.3390/nano13010035

**Published:** 2022-12-22

**Authors:** Antonia Kagkoura, Christina Stangel, Raul Arenal, Nikos Tagmatarchis

**Affiliations:** 1 Theoretical and Physical Chemistry Institute, National Hellenic Research Foundation, 48 Vassileos Constantinou Avenue, 11635 Athens, Greece; 2 Laboratorio de Microscopias Avanzadas (LMA), Universidad de Zaragoza, Mariano Esquillor s/n, 50018 Zaragoza, Spain; 3 Instituto de Nanociencia y Materiales de Aragon (INMA), CSIC-U. de Zaragoza, Calle Pedro Cerbuna 12, 50009 Zaragoza, Spain; 4 ARAID Foundation, 50018 Zaragoza, Spain

**Keywords:** molybdenum diselenide, tungsten diselenide, cobalt-porphyrin, functionalization, hydrogen evolution reaction

## Abstract

Easy and effective modification approaches for transition metal dichalcogenides are highly desired in order to make them active toward electrocatalysis. In this manner, we report functionalized molybdenum diselenide (MoSe_2_) and tungsten diselenide (WSe_2_) via metal-ligand coordination with pyridine rings for the subsequent covalent grafting of a cobalt-porphyrin. The new hybrid materials were tested towards an electrocatalytic hydrogen evolution reaction in both acidic and alkaline media and showed enhanced activity compared to intact MoSe_2_ and WSe_2_. Hybrids exhibited lower overpotential, easier reaction kinetics, higher conductivity, and excellent stability after 10,000 ongoing cycles in acidic and alkaline electrolytes compared to MoSe_2_ and WSe_2_. Markedly, MoSe_2_-based hybrid material showed the best performance and marked a significantly low onset potential of −0.17 V vs RHE for acidic hydrogen evolution reaction. All in all, the ease and fast modification route provides a versatile functionalization procedure, extendable to other transition metal dichalcogenides, and can open new pathways for the realization of functional nanomaterials suitable in electrocatalysis.

## 1. Introduction

Transition metal dichalcogenides (TMDs) have taken centerstage during the past decade due to their interesting properties stemming from their two-dimensional structure [1]. However, a key challenge remains the adaptation of versatile functionalization routes for TMDS en route to the preparation of functional materials, especially efficiently performing in electrocatalysis. TMDs are great alternatives to expensive and finite-source platinum-based electrocatalysts, due to their low-cost, earth abundance, and intrinsic activity toward hydrogen evolution reaction (HER) [1]. Specifically, metallic TMDs (1T type) show intrinsic reduced charge-transfer resistance compared to semiconducting (2H type) ones, which is favorable in electrocatalysis. Interestingly, transition metal diselenides are most understudied among the TMDs family, compared to molybdenum disulfide (MoS_2_), with tungsten diselenide (WSe_2_) being less explored compared to its Mo counterpart molybdenum diselenide (MoSe_2_). This is quite odd since it seems that heavier chalcogens provide better electrical conductivity, which is critical for energy applications [2,3] while tungsten is more affordable and has a more benign nature in comparison with its molybdenum correspondent [4]. TMDs combined with other species, [5,6,7,8,9,10,11] or creating interesting architectures [12,13,14] have shown increased electrocatalytic activity in acidic and alkaline media, while hybridization with a free porphyrin [15] and most importantly with metalloporphyrins [16] provides a favorable way for further improving HER. As porphyrins are macrocyclic tetradentate ligands, they can bind a plethora of metal ions that are active in HER, such as Co, Mn, Ni, and so on. However, high-performing electrocatalysts in acidic media are not efficient in alkaline solutions due to the higher needed overpotentials to initiate the reaction. HER in an alkaline medium is a point of attention due to the less corrosive conditions and low cost but suffers from sluggish reaction kinetics due to the additional water dissociation step [17]. Additionally, hybridization of transition metal diselenides with metalloporphyrins is scarce, [18] while with WSe_2_ has not yet been realized.

Preparation of TMDs by bottom-up approaches involves heating at high temperatures in wet media, followed by the decomposition of target molecules to generate the desired nanocrystals. This kind of crystal growth is easy and leads to large quantities of TMDs having lots of defects and exposed edges favoring electrocatalytic HER [7]. MoS_2_ is the most examined analogue of TMDs and has been extensively used as a model for the study of chemical modification of TMDs through either covalent or non-covalent approaches [19,20]. Covalent functionalization exerts certain assets compared to non-covalent, in terms of the versatility endowed by the variety of functional groups [21,22,23,24] and the efficiency of electronic communication at the functional groups/TMDs interface, both of them being essential for the realization of robust TMD-based electrocatalysts. Sulfur vacant sites at the edges of MoS_2_ are capitalized for the functionalization of both polymorphs, [25,26] whilst for chemically exfoliated TMDs functionalization mostly rests on electron-rich chalcogen species at the basal plane [27,28,29,30,31]. However, functionalization with organic halides has been achieved only to chemically exfoliated 1T-MoS_2_ [31] leading to partial neutralization of negative charges [27] from the surface of 1T-MoS_2_, whereas treatment with aryl diazonium salts has led to complete neutralization, [27] being an unwanted result for electrocatalysis. Thus, robust design for the incorporation of functionalities onto various TMDs with trigonal or octahedral coordination, enabling the integration of species, can give rise to the development of smart nanomaterials with tailor-made physicochemical and electrocatalytic properties.

In this work, we prepared hybrid materials consisting of 1T-MoSe_2_ and cobalt(II) porphyrin (CoP) and 1T-WSe_2_ with CoP for the first time, while their electrocatalytic activity against HER was evaluated. An easy functionalization route was employed to modify MoSe_2_ and Wse_2_ with pyridine moieties through coordination. This novel modification approach can apply to TMDs of both polymorphs towards the realization of novel functional 2D nanomaterials. The newly prepared hybrids were tested as electrocatalysts against HER in alkaline and acidic media, giving high electrocatalytic performance and excellent stability and durability.

## 2. Materials and Methods

**General.** All chemicals, reagents, and solvents were purchased from Sigma-Aldrich (Burlington, MA, USA) and used without further purification unless stated otherwise.

**Characterization techniques**. Raman measurements were acquired with a Renishaw in Via Raman spectrometer, equipped with a CCD camera and a Leica microscope at 514 nm. Renishaw Wire and Origin software were used to record and analyse the data, respectively. Mid-infrared spectra, in the region of 500–4500 cm^−1^ were obtained on a Fourier Transform IR spectrometer (Equinox 55 from Bruker Optics (Ettlingen, Germany)) equipped with a single reflection diamond ATR accessory (DuraSamp1IR II by SensIR Technologies (Danbury, CT, USA)). Scanning transmission electron microscopy (STEM) imaging and electron energy loss spectroscopy (EELS) techniques have been developed using a probe-corrected Titan low-base (Thermo Fisher Scientific, Waltham, MA, USA) working at 120 kV. This microscope is equipped with a high-brightness field-emission gun (X-FEG) and with a Gatan Image Filter (GIF) Tridiem ESR 866 spectrometer for EELS acquisitions. XPS data were recorded using a Kratos Axis Supra spectrometer equipped with a monochromated Al Kα X-ray source using an analyzer pass energy of 160 eV for survey spectra and 20 eV for the core level spectra. Spectra were recorded by setting the instrument to the hybrid lens mode and the slot mode providing approximately a 700 x 300 µm^2^ analysis area using charge neutralization. Regions have been calibrated using the reference value BE(C 1s sp2) = 284.5 eV. All XPS spectra were analyzed using CASA XPS software. The XPS peaks were fitted to GL(70) Voigt lineshape (a combination of 70% Gaussian and 30% Lorentzian character), after performing a Shirley background subtraction. TGA Q500 V20.2 Build 27 instrument by TA in nitrogen (purity > 99.999%) inert atmosphere was used for thermogravimetric analysis.

**Electrochemical measurements**. Autolab PGSTAT128N potentiostat/galvanostat was used for the electrochemical measurements. A standard three-compartment electrochemical cell was used equipped with an RDE with a glassy carbon disk (geometric surface area: 0. 196 cm^2^) as a working electrode, graphite rod as a counter-electrode, and Hg/HgSO_4_ (0.5 M K_2_SO_4_) or Hg/HgO (0.1 M KOH) as reference electrodes. HER LSV measurements were performed in N_2_-saturated aqueous 0.5 M H_2_SO_4_ solution or 0.1 M KOH at room temperature. For preparing the catalyst ink, catalytic powder (4.0 mg) was dissolved in a mixture (1 mL) of deionized water, isopropanol, and 5% Nafion (*v*/*v*/*v* = 4:1:0.02) followed by sonication for 30 min before use. The working electrode was polished with 1, 3, and 6 mm diamond pastes, washed with deionized water, and finally sonicated in double-distilled water before casting 8.5 μL aliquots of the electrocatalytic ink on the electrode’s surface. Finally, electrochemical impedance spectroscopy (EIS) measurements were acquired from 10^5^ to 10^−^^1^ Hz with an AC amplitude of 0.01 V.

**Preparation of WSe_2_.** Tungsten hexacarbonyl (3 mmol) and selenium powder (5.8 mmol) were dissolved in dry p-xylene (145 mL) and the resulting suspension was transferred into a 300 mL Teflon-lined stainless-steel autoclave reactor and heated at 250 °C for 24 h. After the autoclave was cooled to room temperature, the resulting suspension was filtrated through a PTFE membrane filter (0.2 μm) to remove unreacted species and rinsed with acetone and dichloromethane to remove unreacted species to obtain WSe_2_ (790 mg).

**Preparation of MoSe_2_.** Molybdenum hexacarbonyl (3 mmol) and selenium powder (5.8 mmol) were dissolved in dry p-xylene (145 mL) and the mixture was transferred to a 300 mL Teflon-lined stainless-steel autoclave reactor and held at 250 °C for 24 h. The resulting suspension was filtrated through a PTFE membrane filter (0.2 μm) to remove unreacted species and rinsed with acetone and dichloromethane to get MoSe_2_ (810 mg).

**Preparation of f-WSe_2_.** In a round bottom flask, WSe_2_ (30 mg) was dispersed in H_2_O (30 mL). After sonication, 4-aminopyridine (0.3375 mmol) was added to the mixture and sonicated for 60 s. The resulting suspension was filtrated through a PTFE membrane filter (0.2 μm) and rinsed with water to obtain f-WSe_2_ (48 mg).

**Preparation of f-MoSe_2_.** In order to obtain f-MoSe_2_, a similar procedure was followed.

**5-(4-carboxyphenyl)-10,15,20-triphenylporphyrinCo(II).** CoCl_2_ (54 mg, 0.22 mmol), 5-(4-carboxyphenyl)-10,15,20-triphenylporphyrin (10 mg, 0.015 mmol) and DMF (5 mL) were added in a round bottom flask and the reaction mixture was heated to reflux for 4 h. After, DMF was distilled; the residue was dissolved in a mixture of CH_2_Cl_2_ together with two drops of methanol. The organic layer was washed two times with water and dried over Na_2_SO_4_ followed by filtration and evaporation. Finally, column chromatography (SiO_2_, CH_2_Cl_2_/MeOH, 100:4) and reprecipitation of the product with CH_2_Cl_2_ and cold hexane furnished CoP as a dark red-brown solid (9 mg, 83%). The ^1^H-NMR spectrum showed broad peaks due to the presence of paramagnetic Co(II). FT-IR: ṽ = 1690 (C=O carboxylic), 1604 (C=C), 1354 (C-N), 1070 (C-H), 704 (N-H) cm^−1^ (porphyrin core vibrations). UV-Vis (CH_2_Cl_2_): λmax, abs= 410, 528 nm.

**Preparation of WSe_2_-CoP.** CoP (7.3 mg) was added to dry dichloromethane (15 mL) and sonicated for 30 min under an N_2_ atmosphere. Next, EDCI (13.42 mg, 0.07 mmol) was added at 0 °C and the mixture was left to stir for 1 h, followed by the addition of f-WSe_2_ (8 mg), HOBt (13.51 mg, 0.1 mmol), and N,N-diisopropylethylamine (20 μL). The dispersion was then left to stir for five days at room temperature. Finally, the resulting suspension was filtrated through a PTFE membrane filter (0.2 μm) and rinsed with dichloromethane, methanol and acetone to obtain 15 mg of WSe_2_-CoP.

**Preparation of MoSe_2_-CoP.** A similar procedure was followed for the preparation of MoSe_2_-CoP.

## 3. Results and Discussion

**Figure 1:**
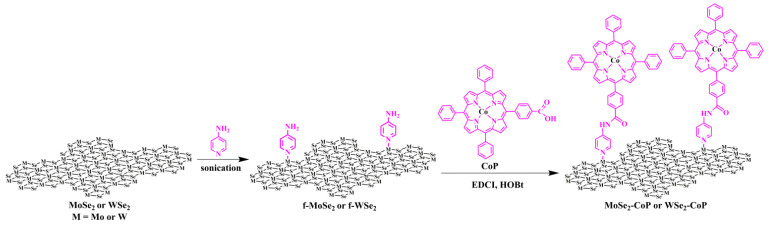
Synthetic illustration for the functionalization of MoSe_2_ and WSe_2_ and subsequent preparation of MoSe_2_-CoP and WSe_2_-CoP.

Firstly, MoSe_2_ and WSe_2_ were prepared by a one-pot reaction in an autoclave [32]. Heating of molybdenum hexacarbonyl (Mo(CO)_6_) (tungsten hexacarbonyl (W(CO)_6_) alternatively) and Se powder in p-xylene produced grams of 1T-MoSe_2_ (and 1T-WSe_2_, respectively). Afterward, sonication (ca. 1 min.) of the aqueous dispersion of TMDs and aminopyridine yielded amino terminated groups to the transition metal diselenides, via the formation of a metal-ligand bond between Mo or W and the pyridinic nitrogen, furnishing functionalized MoSe_2_ (f-MoSe_2_) and WSe_2_ (f-WSe_2_), respectively. Next, a condensation reaction between amino-modified transition metal diselenides and carbonyl-terminated CoP led to the formation of MoSe_2_-CoP and WSe_2_-CoP, as seen in Figure 1.

**Figure 2:**
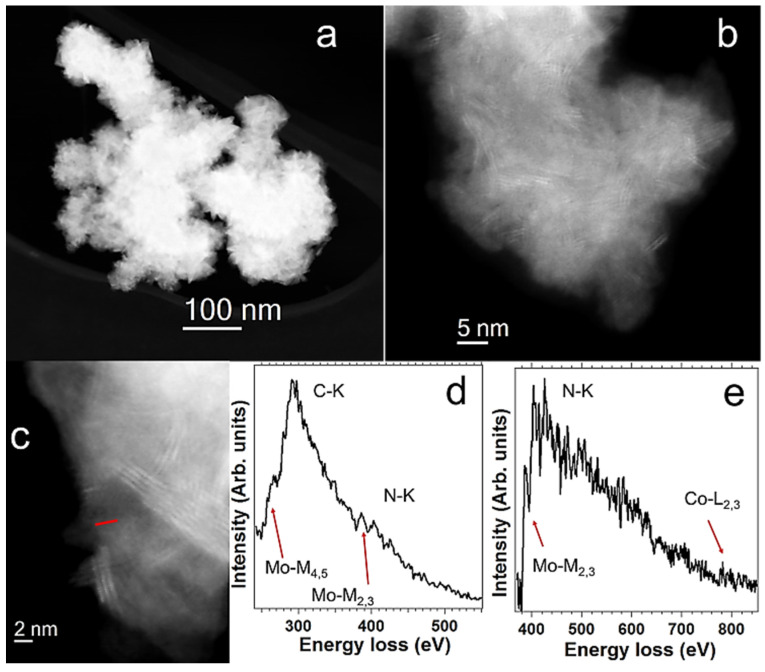
Scanning transmission electron microscopy (STEM) analyses. (**a**–**c**) High-angle annular dark field (HAADF) STEM images of a MoSe_2_-CoP flake, where the 1T phase can be identified (**b**,**c**). (**d**,**e**) An EELS spectrum-line is recorded in the highlighted area of (**c**). 15 EEL spectra, recorded in the red-marked line of the (**c**), were selected and added. Two different energy regions are displayed, where different elements and absorption edges can be observed, see (**d**,**e**). These elements correspond to MoSe_2_ (Mo-M edge) and to the CoP moieties (C-K, N-K and Co-L edges).

The morphology, structure, and chemical composition of these hybrids were analyzed via scanning transmission electron microscopy (STEM). Figure 2a shows that flakes of around one micron were obtained. They are similar to the ones of the starting MoSe_2_ material (Figure S1). High-resolution high-angle annular dark field (HAADF) STEM images of these flakes of MoSe_2_-CoP show that the TMD material corresponds to the 1T phase (Figure 2b,c). Similar STEM-HAADF imaging analyses were also performed on WSe_2_-CoP (Figure S2). Electron energy-loss spectroscopy (EELS) in the spectrum-line model [33,34] was employed to locally investigate the chemical nature of these nanostructures and in particular to identify the CoP moieties. An EELS spectrum-line was recorded in the highlighted area of Figure 2c. From this EELS spectrum-line, 15 EEL spectra, recorded in the red-marked line of Figure 2c, were selected and added. Two different energy regions of the EEL spectra are displayed in Figure 2d,e. The detection of C, N, and Co in this area confirms the presence of the CoP entities at the surface of MoSe_2_.

**Figure 3:**
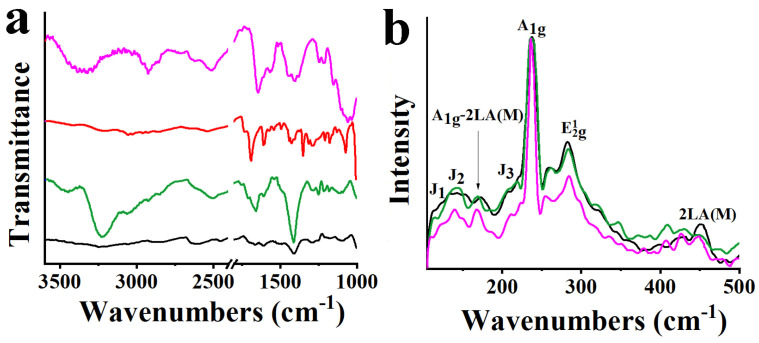
(**a**) ATR-IR and (**b**) Raman spectra (514 nm) for MoSe_2_-CoP (pink), MoSe_2_ (black), f-MoSe_2_ (green), and CoP (red).

In order to follow the modification that occurred on MoSe_2_ and WSe_2_, with the ultimate goal to verify the successful formation of MoSe_2_-CoP and WSe_2_-CoP, diverse spectroscopic, thermal, and microscopy imaging techniques were employed. For starters, typical bands of pyridine rings are seen in the IR spectrum of f-MoSe_2_ due to the successful coordination of the former at the metal of MoSe_2_, at 3430 (N-H), 1651 (C-N) and 1411 cm^−^^1^ (C-H (Figure 3a). On the other hand, CoP shows the carbonyl vibration mode at 1690 cm^−1^, due to the surface -COOH units. The IR spectrum of MoSe_2_-CoP is occupied by the characteristic band at 1641 cm^−^^1^ owned to the carbonyl amide stretching due to the successful hybridization, while N-H vibrational features are evident in MoSe_2_-CoP. In a similar manner, the effective grafting of amino groups onto WSe_2_ is obvious by the characteristic bands due to the pyridine ring at 3430 (N-H), 1646 and 1329 (C-N), and 1403 cm^−^^1^ (C-H) (Figure S3a). Additionally, the coupling of amino-modified WSe_2_ with the carbonyl terminated CoP is shown at 1650 cm^−^^1^, due to the carbonyl amide stretching, as seen in Figure S3a.

Next, Raman spectra were recorded upon excitation at 514 nm. Specifically, the spectrum of MoSe_2_ is guided by the A_1g_-LA(M), A_1g_, E^1^_2g_, and 2LA(M) bands at 171, 238, 285, and 450 cm^−^^1^ respectively. Meanwhile, bands located at 118, 137, and 223 cm^−^^1^ correspond to J_1_, J_2,_ and J_3_ phonon modes, respectively, and are indicative of the 1T octahedral phase of MoSe_2_ [35]. In the spectrum of f-MoSe_2_, the suppression of the 2LA(M) band, which is associated with chalcogen vacancies and defect sites, compared to MoSe_2_ is indicative of the metal-ligand coordination that took place at the defect sites. The fact that there is no alteration in the intensity of the 2LA(M) mode in MoSe_2_-CoP indicates that the covalent grafting of CoP onto MoSe_2_ does not affect the basal plane of MoSe_2_ (Figure 3b). Accordingly, the spectrum of WSe_2_ shows apparent prominent peaks at 175, 237, and 256 cm^−^^1^ corresponding to A_1g_-LA(M), E^1^_2g_, A_1g_, and 2LA(M) modes, while E^1^_2g_, and A_1g_ modes overlap [36,37,38]. Additional J_1_, J_2_, and J_3_ bands located at 112, 148, and 216 cm^−^^1^, respectively, are fingerprint phonon modes of the metallic phase of WSe_2_ [34]. As mentioned previously, the decrease in the intensity of the 2LA(M) band of f-WSe_2_ reveals the successful functionalization, while intensity remained the same for WSe_2_-CoP (Figure S3b).

**Figure 4:**
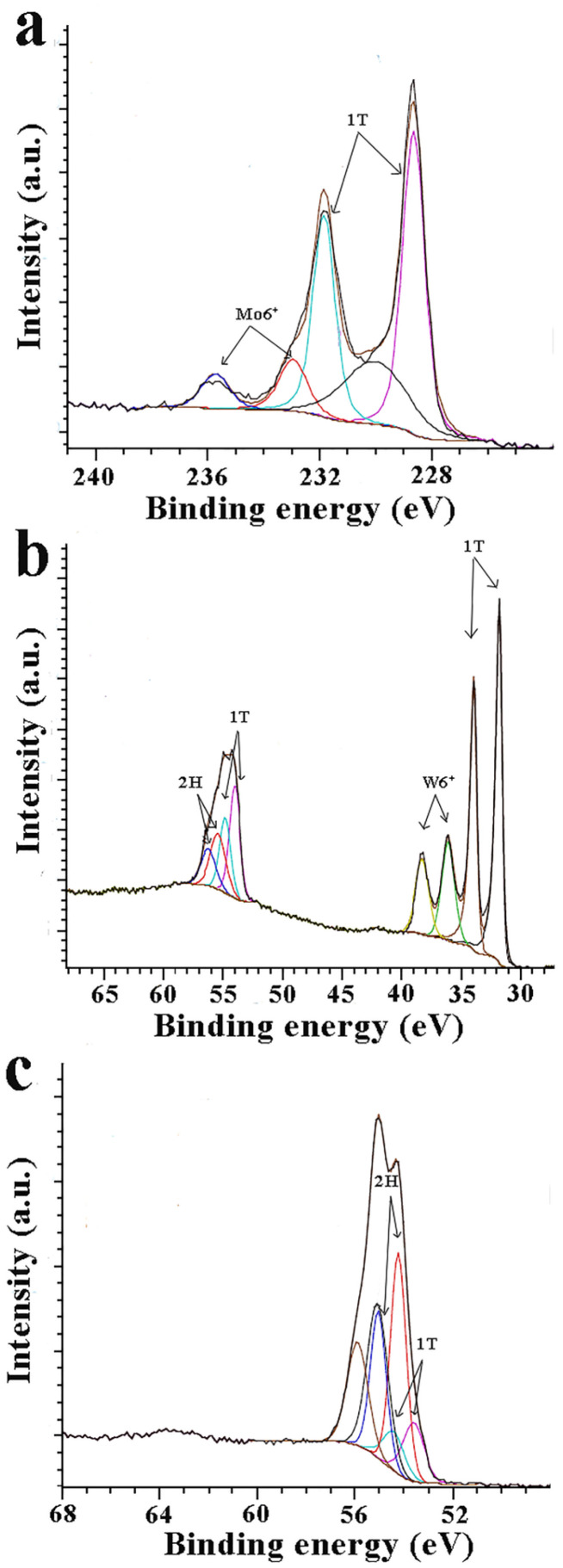
Deconvoluted X-ray photoelectron spectra of (**a**) Mo 3d for MoSe_2_, (**b**) W 4f and Se 3d for WSe_2_, and (**c**) Se 3d for MoSe_2_.

Insight into the MoSe_2_ and WSe_2_ phases was given from X-ray photoelectron spectroscopy (XPS) measurements. Figure 4a depicts the deconvoluted Mo 3d spectra, showing doublets at 228.6 eV for Mo 3d_5/2_ and 231.8 eV for Mo 3d_3/2_, revealing the existence of the 1T phase in MoSe_2_. The doublet at higher energies, 232.9 eV for 3d_5/2_ and 235.7 for 3d_3/2_, corresponds to Mo^6+^ [39,40]. Similarly, deconvoluted W 4f spectra are shown in Figure 4b. The apparent doublet of the 4f W^+4^ peaks at 31.7 for W^4+^ 4f_7/2_ and 33.9 eV for W^4+^ 4f_5/2_ is assigned to the 1T phase, while signals at 36.04 and 38.22 eV indicate the presence of W^6+^ species within WSe_2_ [40,41]. The Se 3d peak profile of MoSe_2_ can be fitted into two sets of doublet peaks at higher energies (55.0 eV for Se 3d_3/2_ and 54.2 eV for Se 3d_5/2_) for the 2H phase and another couple at lower energies (54.4 eV for Se 3d_3/2_ and 53.6 eV for Se 3d_5/2_) for the 1T phase (Figure 4c) [39,40]. The same applies to the Se 3d peak profile of WSe_2_; peaks at 55.3 eV for Se 3d_3/2_ and 56.1 eV for Se 3d_5/2_ for the 2H phase and 54.7 eV for Se 3d_3/2_ and 53.7 eV for Se 3d_5/2_ for the 1T phase (Figure 4a) [40,41]. The above results are aligned with the Raman data suggesting the strong presence of the 1T phase in MoSe_2_ and WSe_2_.

**Figure 5:**
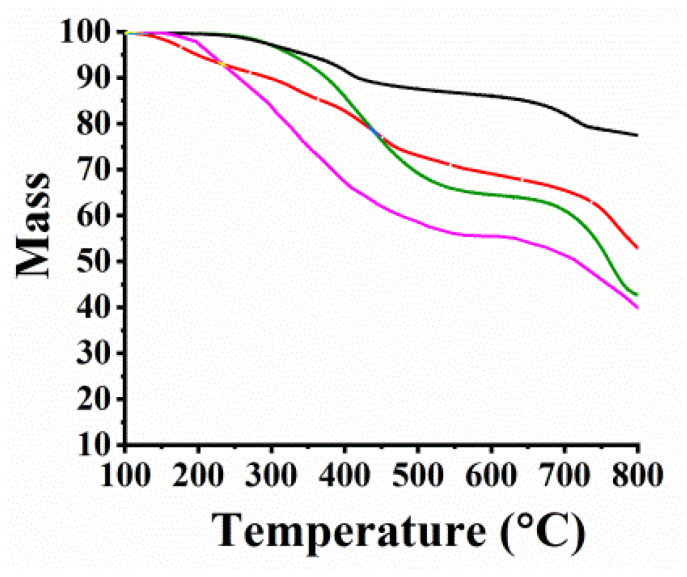
TGA graphs for MoSe_2_-CoP (pink), MoSe_2_ (black), f-MoSe_2_ (green), and CoP (red).

Thermogravimetric analysis (TGA) was employed to gather information about the thermal stability of the hybrid materials under an N_2_ atmosphere. It can be seen in Figure 5 that MoSe_2_ losses an 14% mass up to 550 °C, attributed to the generation of defects during the solvothermal preparation (Figure 5). A higher mass loss of up to 21% is shown for the f-MoSe_2_ up to the same temperature region, revealing the thermal decomposition of the incorporated organic species onto MoSe_2_. Finally, MoSe_2_-CoP presents an additional mass loss of 9% due to the successful grafting of CoP to f-MoSe_2_. Rest on the above, the loading in the MoSe_2_-CoP material was calculated to be one CoP for every 16 f-MoSe_2_ units. Furthermore, Figure S4 shows the thermographs of WSe_2_-based materials. Accordingly, the loading in the WSe_2_-CoP was calculated to be one CoP for every 16 f-WSe_2_ units.

**Figure 6:**
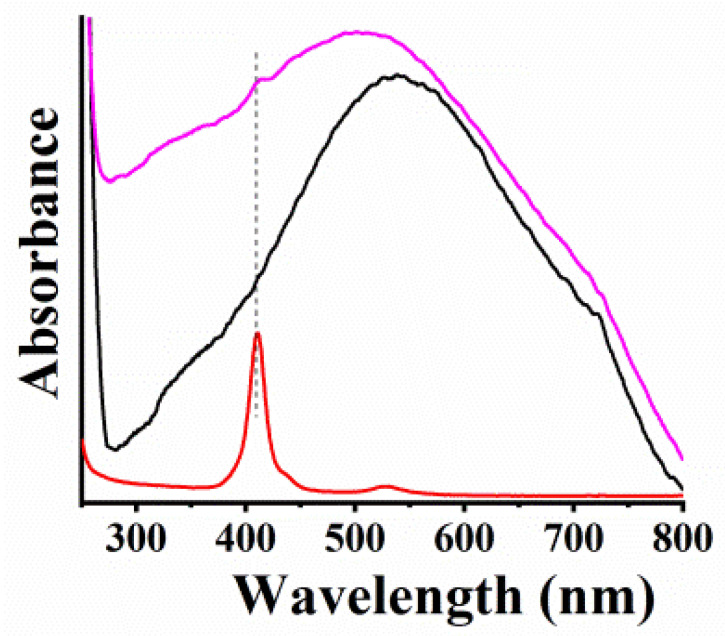
UV-Vis spectra for MoSe_2_-CoP (pink), MoSe_2_ (black), and CoP (red), in dichloromethane.

Steady-state electronic absorption spectroscopy provided further proof for the formation of MoSe_2_-CoP and WSe_2_-CoP (Figure 6 and Figure S5, respectively). The absorption spectra for intact MoSe_2_ and WSe_2_ exhibit strong and broad absorption in the visible region without any distinct features, typical for TMDs of 1T-phase derived from bottom-up approaches [36,37]. Meanwhile, in the absorption spectrum of MoSe_2_-CoP, the Soret band at 410 nm is evident, also visible in the UV-Vis spectrum of CoP (Figure 6), whereas the band of MoSe_2_ centered at 537 nm is blue-shifted at 502 nm indicating the electronic communication of MoSe_2_ and CoP within MoSe_2_-CoP hybrid at the ground state. Moreover, the UV-Vis spectrum of WSe_2_-CoP (Figure S5) shows a distinct absorption band at 410 nm, deriving from the successful conjugation of f-WSe_2_ with CoP.

**Figure 7:**
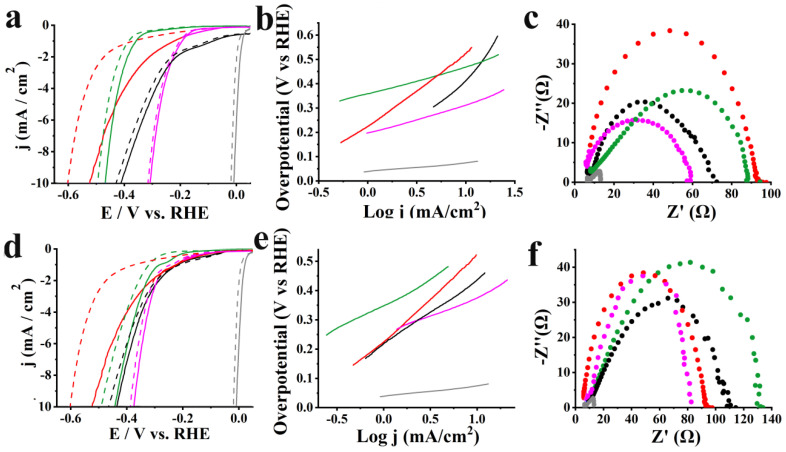
(**a**) LSVs for HER obtained at 1600 rpm rotation speed and 5 mV/s scan rate before (solid lines) and after 10,000 cycles (dashed lines) in aqueous 0.5 Μ H_2_SO_4_, (**b**) Tafel slopes, and (**c**) Nyquist plots for MoSe_2_-CoP (pink), MoSe_2_ (black), f-MoSe_2_ (green), CoP (red) and Pt/C (grey). (**d**) LSVs for HER obtained at 1600 rpm rotation speed and 5 mV/s scan rate before (solid lines) and after 10,000 cycles (dashed lines) in 0.5 Μ H_2_SO_4_, (**e**) Tafel slopes, and (**f**) Nyquist plots for WSe_2_-CoP (pink), WSe_2_ (black), f-WSe_2_ (green), CoP (red) and Pt/C (grey).

Next, electrocatalytic acidic HER for hybrids and reference materials was evaluated by recording linear sweep voltammetry (LSV). Notably, significant low onset potential was recorded for MoSe_2_-CoP at −0.17 V vs RHE, lower than those recorded for MoSe_2_, f-MoSe_2_, and CoP at −0.22, −0.35 and −0.28 V vs RHE, respectively (Figure 7a). The beneficial role of CoP was further revealed by evaluating the HER at −10 mA/cm^2^. In fact, MoSe_2_-CoP operates HER at −10 mA/cm^2^ at −0.31 V vs RHE, ca. 100 mV lower than that of MoSe_2_, at −0.41 V vs RHE. For f-MoSe_2_ and CoP, higher onset potential values are noted at −0.47 and −0.52 V, respectively. At the same time, hydrogen evolution for WSe_2_-CoP starts at lower potentials, at −0.22 V vs RHE compared to reference materials, while the lowest potential value was required to drive HER at a reference current density of −10 mA/cm^2^, at −0.33 V i.e., 100 mV lower compared to WSe_2_ (Figure 7d). The significantly lower overpotential value for driving protons reduction to molecular hydrogen for hybrid materials is justified by the presence of CoP, while the covalent grafting between CoP and MoSe_2_ and/or WSe_2_ promotes charge transfer and current flow driving the overall reaction to lower potentials.

Tafel slope values were extracted to gain information on the reaction mechanism. In acidic HER protons are initially adsorbed onto the electrode surface via a reduction process (Volmer step). Afterward, the desorption of adsorbed hydrogen atoms onto the electrode (Heyrovsky step) or the recombination of two adsorbed protons (Tafel step) follows generating molecular H_2_. With all that said, the smooth current flow within the MoSe_2_-CoP is confirmed by the lower Tafel slope value of 114 mV/dec, compared to the higher Tafel slope value of MoSe_2_ (375 mV/dec), f-MoSe_2_ (123 mV/dec) and CoP (288 mV/dec), suggesting that the release of the molecular hydrogen onto the electrode is the rate-limiting step (Figure 7b). The same applies to WSe_2_-CoP which displays the lowest Tafel slope value (133 mV/dec) among Wse_2_-based electrocatalysts (Figure 6e).

Additional insight into HER kinetics is obtained by electrochemical impedance spectroscopy (EIS) assays. In Figure 7c, MoSe_2_-CoP shows the smaller frequency semicircle in the Nyquist plot compared to MoSe_2_ and f-MoSe_2_, corresponding to a smaller charge transfer resistance (R_ct_) value of 53 Ω than the much higher R_ct_ values of ca. 63, 74 and 84 Ω for MoSe_2_, f-MoSe_2_, and CoP respectively. Similarly, WSe_2_-CoP shows a lower R_ct_ value of 75 Ω compared to WSe_2_ (92 Ω) and f-WSe_2_ (119 Ω), (Figure 7f). The lower R_ct_ values for hybrid materials reflect higher conductance and more facile electron transfer kinetics in MoSe_2_-CoP and WSe_2_-CoP due to the covalent linkage of the TMDs with the cobalt-metallated porphyrin. The stability of hybrids was assessed, after performing 10,000 ongoing electrocatalytic cycles in acidic media as shown in Figure 7a and Figure 6d, respectively. Interestingly, MoSe_2_-CoP and WSe_2_-CoP exhibited extremely high stability as they showed negatively shifted LSV curves of only 10 mV after continuous cycling. Table S1 sums up the electrocatalytic acidic HER data before and after 10,000 cycles for hybrids and reference materials and Pt/C.

The superior electrocatalytic performance of MoSe_2_-CoP and WSe_2_-CoP was also observed towards alkaline HER, as seen in Figure S6a,d, respectively, as hybrid materials note significantly lower overpotentials compared to reference materials. Additionally, lower Tafel slope values were calculated for MoSe_2_-CoP (Figure S6b) and WSe_2_-CoP (Figure S6d) along with high R_ct_ values, Figure S6c,f, respectively, reflecting the easier current flow within the hybrids. However, it should be noted that acidic HER is less complicated compared to alkaline HER. In the former case, the reduction of hydronium ions (H_3_O^+^) to gaseous dihydrogen (H_2_) occurs during water electrolysis, while in alkaline HER extra energy is needed to produce the protons by breaking the water molecule, affecting the overall reaction rates [42]. Additionally, in alkaline HER Volmer and Heyrovsky steps are likely to include a water-dissociation step [43] due to the vast decrease in proton concentration as detailed below:

H_2_O + e^−^ = H* + OH^−^ (Volmer step)

H_2_O + e^−^ + H* = H_2_ + OH^−^ (Heyrovsky step)

Indeed, the above are depicted in the higher overpotentials and Tafel slope values compared to acidic HER (Table S1). Finally, the higher stability for both electrocatalysts is shown in Figure S6a,d, while relative data are summarized in Table S1.

All in all, the advantageous role of CoP in electrocatalytic HER is depicted in both acidic and alkaline media within the hybrid materials. MoSe_2_-CoP and WSe_2_-CoP show improved performance compared to just MoSe_2_ and WSe_2_ as they achieve lowered overpotentials against HER, possess easier reaction kinetics and improved conductivity featuring the beneficial presence of CoP along with the effective conjugation with the TMDs.

## 4. Conclusions

The adaptation and exploration of versatile modification routes for TMDs toward the realization of novel electrocatalysts is a hot issue. In this work, we have demonstrated an easy and fast functionalization approach, via a metal-ligand covalent bond, for the introduction of amino-terminated pyridine rings onto MoSe_2_ and WSe_2_ and the subsequent covalent linkage with carbonyl-terminated cobalt(II) porphyrins. The new hybrids exhibited improved HER activity in acidic and alkaline media and showed easier reaction kinetics, higher conductivity, and stability compared to bare transition metal diselenides. Noticeably, MoSe_2_-CoP showed the best performance and reached a low onset potential of −0.17 V vs RHE towards acidic HER. The successful incorporation of cobalt-porphyrin onto MoSe_2_ and WSe_2_ speeded up the initiation of HER, while covalent linkage improved charge delocalization and transfer in neighboring species leading to high electrocatalytic activity for the reduction of protons to molecular hydrogen. Finally, the facile functionalization approach can be applied for the modification of other transition metals dichalcogenides and may open new routes for the realization and exploration of functional electrocatalysts.

## Data Availability

Not applicable.

## Supplementary Materials

The following supporting information can be downloaded at: https://www.mdpi.com/article/10.3390/nano13010035/s1, Figure S1: (a,b) HAADF-STEM images of a MoSe_2_ flake.; Figure S2: (a,b) HAADF-STEM images of a WSe_2_-CoP flake; Figure S3: (a) ATR-IR, and (b) Raman spectra of WSe_2_-CoP (pink), WSe_2_ (black), f-WSe_2_ (green), and CoP (red); Figure S4. TGA graphs for WSe_2_-CoP (pink), WSe_2_ (black), f-WSe_2_ (green), and CoP (red); Figure S5. UV-Vis spectra for WSe_2_-CoP (pink), WSe_2_ (black), and CoP (red), in dichloromethane; Figure S6: (a)LSVs for HER obtained at 1600 rpm rotation speed and 5 mV/s scan rate before (solid lines) and after 10,000 cycles (dashed lines) in aqueous 0.1 KOH, (b) Tafel slopes and (c) Nyquist plots for materials MoSe_2_-CoP (pink), MoSe_2_ (black), f-WSe_2_ (blue), CoP (red) and Pt/C (grey). (d)LSVs for HER obtained at 1600 rpm rotation speed and 5 mV/s scan rate before (solid lines) and after 10,000 cycles (dashed lines) in aqueous 0.1 KOH, (e) Tafel slopes and (f) Nyquist plots for materials WSe_2_-CoP (pink), WSe_2_ (black), f-WSe_2_ (green), CoP (red) and Pt/C (grey); Table S1: Electrocatalytic HER parameters for MoSe_2_-CoP and WSe_2_-CoP in comparison with materials MoSe_2_, WSe_2_, f-MoSe_2_, f-WSe_2_, CoP and Pt/C.
