# Supplementary material for: Molybdenum Diselenide and Tungsten Diselenide Interfacing Cobalt-Porphyrin for Electrocatalytic Hydrogen Evolution in Alkaline and Acidic Media
Source: Nanomaterials (Basel). 2022 Dec 22;13(1):35. doi: 10.3390/nano13010035 (PMC9824367; doi:10.3390/nano13010035)
Supplement: Supplementary file 1 [file nanomaterials-13-00035-s001.zip › nanomaterials-2089665-supplementary.pdf]

Electronic Supporting Information

Molybdenum Diselenide and Tungsten Diselenide Interfacing Cobalt-Porphyrin for Electrocatalytic Hydrogen Evolution in Alkaline and Acidic Media

Antonia Kagkoura [1,*], Christina Stangel [1], Raul Arenal [2,3,4,*] and Nikos Tagmatarchis [1,*]

1 Theoretical and Physical Chemistry Institute, National Hellenic Research Foundation, 48 Vassileos Constantinou Avenue, 11635 Athens, Greece
2 Laboratorio de Microscopias Avanzadas (LMA), Universidad de Zaragoza, Mariano Esquillor s/n, 50018 Zaragoza, Spain
3 Instituto de Nanociencia y Materiales de Aragon (INMA), CSIC-U. de Zaragoza, Calle Pedro Cerbuna 12, 50009 Zaragoza, Spain
4 ARAID Foundation, 50018 Zaragoza, Spain
* Correspondence: akagkoura@eie.gr (A.K.); arenal@unizar.es (R.A.); tagmatar@eie.gr (N.T.)

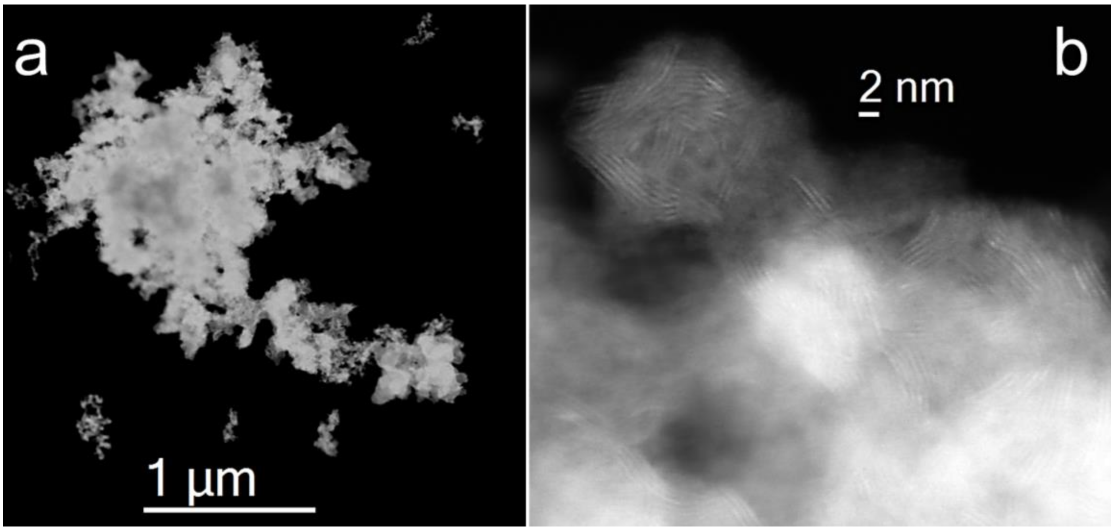

Figure S1. (a, b) HAADF-STEM images of a $MoSe_2$ flake.

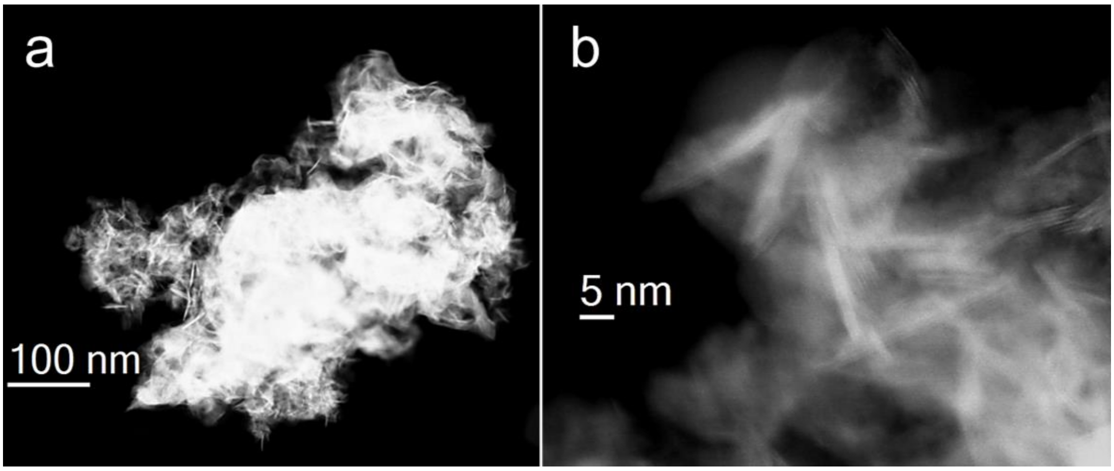

Figure S2. (a, b) HAADF-STEM images of a WSe_2-CoP flake.

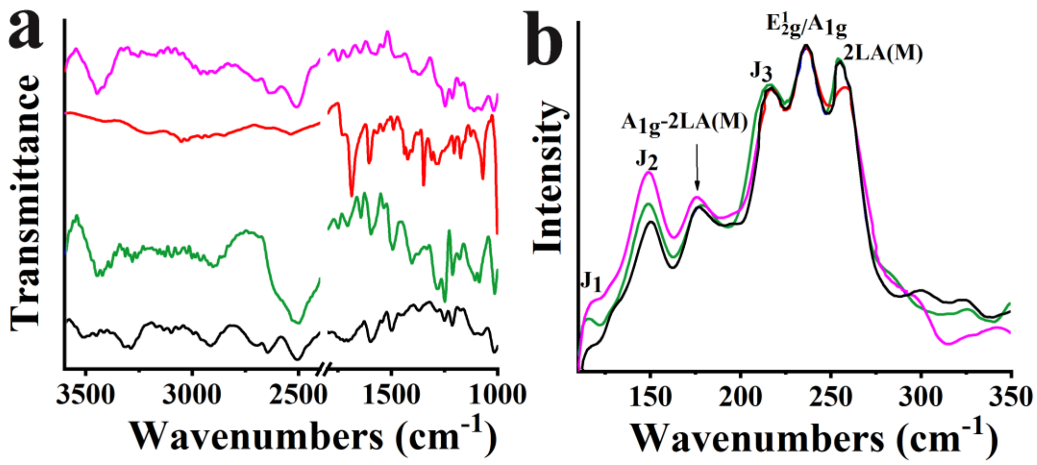

Figure S3. (a) ATR-IR, and (b) Raman spectra of WSe_2-CoP (pink), WSe_2 (black), f-WSe_2 (green), and CoP (red).

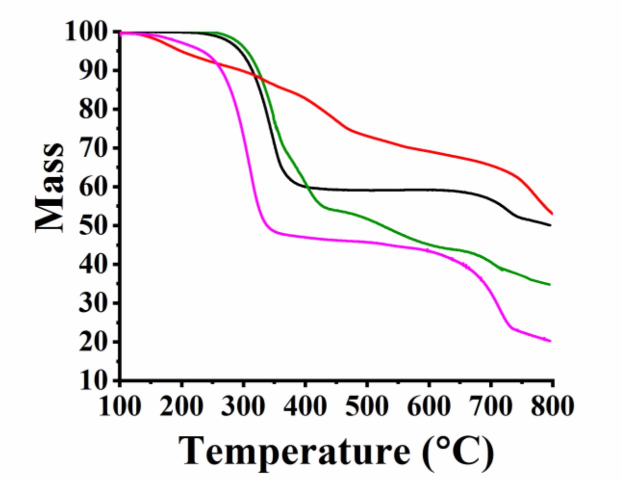

Figure S4. TGA graphs for WSe_2-CoP (pink), WSe_2 (black), f-WSe_2 (green) and CoP (red).

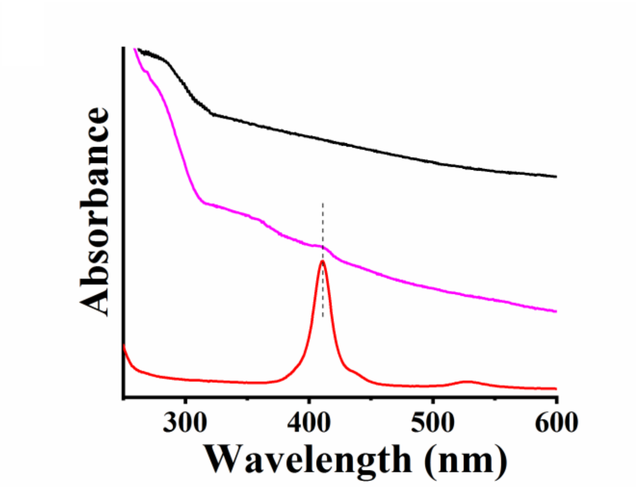

Figure S5. UV-Vis spectra for WSe_2-CoP (pink), WSe_2 (black) and CoP (red), in dichloromethane.

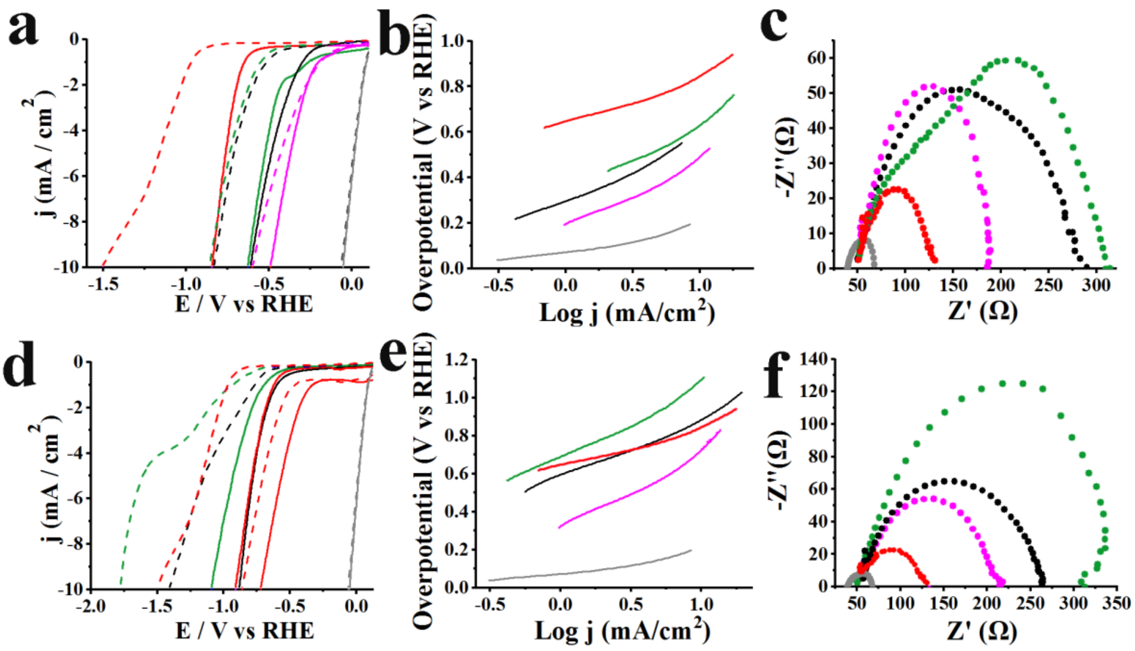

Figure S6. (a)LSVs for HER obtained at 1,600 rpm rotation speed and 5 mV/s scan rate before (solid lines) and after 10,000 cycles (dashed lines) in aqueous 0.1 KOH, (b) Tafel slopes and (c) Nyquist plots for materials $MoSe_2$-CoP (pink), $MoSe_2$ (black), f-WSe_2 (blue), CoP (red) and Pt/C (grey). (d)LSVs for HER obtained at 1,600 rpm rotation speed and 5 mV/s scan rate before (solid lines) and after 10,000 cycles (dashed lines) in aqueous 0.1 KOH, (e) Tafel slopes and (f) Nyquist plots for materials WSe_2-CoP (pink), WSe_2 (black), f-WSe_2 (green), CoP (red) and Pt/C (grey).

Table S1. Electrocatalytic HER parameters for $MoSe_2$-CoP and WSe_2-CoP in comparison with materials $MoSe_2$, WSe_2, f-$MoSe_2$, f-WSe_2, CoP and Pt/C.

Electrocatalyst	Onset potential (V vs RHE)	Potential (V vs RHE) at -10 mA/cm^2	Tafel slope (mV/dec)	R_{ct} (Ω)	Electrolyte
$MoSe_2$-CoP	-0.17	-0.31	114	53	0.5 M H_2SO_4
$MoSe_2$-CoP[a]	-0.19	-0.32	116	-	0.5 M H_2SO_4
$MoSe_2$	-0.22	-0.41	375	63	0.5 M H_2SO_4
$MoSe_2$[a]	-0.24	-0.43	375	-	0.5 M H_2SO_4
f-$MoSe_2$	-0.35	-0.47	123	74	0.5 M H_2SO_4
f-$MoSe_2$[a]	-0.38	-0.49	125	-	0.5 M H_2SO_4
CoP	-0.28	-0.52	288	84	0.5 M H_2SO_4
CoP*	-0.48	-0.60	250	-	0.5 M H_2SO_4
WSe_2-CoP	-0.22	-0.33	133	75	0.5 M H_2SO_4
WSe_2-CoP[a]	-0.29	-0.38	138	-	0.5 M H_2SO_4
WSe_2	-0.28	-0.43	217	92	0.5 M H_2SO_4
WSe_2[a]	-0.31	-0.46	264	-	0.5 M H_2SO_4
f-WSe_2	-0.3	-0.44	200	119	0.5 M H_2SO_4
f-WSe_2*	-0.33	-0.49	180	-	0.5 M H_2SO_4
Pt/C	0.029	-0.009	35	6.1	0.5 M H_2SO_4
Pt/C[a]	0.011	-0.020	35	-	0.5 M H_2SO_4
$MoSe_2$-CoP	-0.22	-0.48	240	138	0.1 M KOH
$MoSe_2$-CoP[a]	-0.22	-0.58	314	-	0.1 M KOH
$MoSe_2$	-0.28	-0.62	290	246	0.1 M KOH
$MoSe_2$[a]	-0.5	-0.83	298	-	0.1 M KOH
f-$MoSe_2$	-0.4	-0.63	340	280	0.1 M KOH
f-$MoSe_2$[a]	-0.55	-0.85	531	-	0.1 M KOH
CoP	-0.64	-0.84	295	74	0.1 M KOH
CoP[a]	-0.95	-1.5	219	-	0.1 M KOH
WSe_2-CoP	-0.35	-0.72	280	173	0.1 M KOH
WSe_2-CoP[a]	-0.49	-0.86	453	-	0.1 M KOH
WSe_2	-0.60	-0.88	350	215	0.1 M KOH
WSe_2[a]	-0.73	-1.41	434	-	0.1 M KOH
f-WSe_2	-0.68	-1.09	390	275	0.1 M KOH
f-WSe_2[a]	-0.94	-1.78	531	-	0.1 M KOH
Pt/C	0.11	-0.053	120	33	0.1 M KOH
Pt/C[a]	0.10	-0.065	122	-	0.1 M KOH

[a]after 10,000 cycles
